# Evaluation of Relevance between Advanced Glycation End Products and Diabetic Retinopathy Stages Using Skin Autofluorescence

**DOI:** 10.3390/antiox9111100

**Published:** 2020-11-09

**Authors:** Yuji Takayanagi, Mikihiro Yamanaka, Jo Fujihara, Yotaro Matsuoka, Yuko Gohto, Akira Obana, Masaki Tanito

**Affiliations:** 1 Department of Ophthalmology, Shimane University Faculty of Medicine, Izumo 693-8501, Japan; y.takayanagi1008@med.shimane-u.ac.jp; 2 Laboratory of Food and Regulation Biology, School of Agriculture, Tokai University, Kumamoto 862-8652, Japan; yamanaka.mikihiro@tsc.u-tokai.ac.jp; 3 Division of Ophthalmology, Matsue Red Cross Hospital, Matsue 690-8506, Japan; aoiroelephant@hotmail.co.jp (J.F.); ymatsu@med.shimane-u.ac.jp (Y.M.); 4 Department of Ophthalmology, Seirei Hamamatsu General Hospital, Hamamatsu 430-8558, Japan; yukogo@sis.seirei.or.jp (Y.G.); obana@sis.seirei.or.jp (A.O.)

**Keywords:** advanced glycation end products (AGEs), skin autofluorescence (sAF), AGEs Sensor, diabetic retinopathy, proliferative diabetic retinopathy, neovascular glaucoma, oxidative stress

## Abstract

Advanced glycation end products (AGEs) are thought to play important roles in the pathogenesis of diabetic microangiopathy, particularly in the progression of diabetic retinopathy (DR). We assessed the levels of skin autofluorescence (sAF) to assess the association between AGEs and DR stages. A total of 394 eyes of 394 Japanese subjects (172 men, 222 women; mean age ± standard deviation [SD], 68.4 ± 13.7 years) comprised the study population, i.e., subjects with diabetes mellitus (DM) (*n* = 229) and non-diabetic controls (*n* = 165). The patients with DM were divided into those without DR (NDR, *n* = 101) and DR (*n* = 128). DR included simple (SDR, *n* = 36), pre-proliferative (PPDR, *n* = 25), and PDR (*n* = 67). Compared to controls (0.52 ± 0.12), the AGE scores were significantly higher in patients with DM (0.59 ± 0.17, *p* < 0.0001), NDR (0.58 ± 0.16, *p* = 0.0012), and DR (0.60 ± 0.18, *p* < 0.0001). The proportion of patients with PDR was significantly higher in the highest quartile of AGE scores than the other quartiles (*p* < 0.0001). Compared to those without PDR (SDR and PPDR), those with PDR were younger (*p* = 0.0006), more were pseudophakic (*p* < 0.0001), had worse visual acuity (VA) (*p* < 0.0001), had higher intraocular pressure (IOP) (*p* < 0.0001), and had higher AGE scores (*p* = 0.0016). Multivariate models also suggested that younger age, male gender, pseudophakia, worse VA, higher IOP, and higher AGE scores were risk factors for PDR. The results suggested that AGE scores were higher in patients with DM and were independently associated with progression of DR. In addition, more PDR was seen in the highest quartile of AGE scores. This study highlights the clinical use of the AGE score as a non-invasive, reliable marker to identity patients at risk of sight-threatening DR.

## 1. Introduction

Diabetic retinopathy (DR), which is a major vascular complication of diabetes mellitus (DM), has been recognized for decades, and disease progression often results in devastating visual loss [1]. Although several previous studies have shown that hyperglycemia, smoking, hypertension, and other factors are related closely to diabetic angiopathy [2,3,4,5], these traditional factors do not fully explain the development of the vascular complications of DM and the involvement of other factors likely to promote this process.

The accumulation of advanced glycation end products (AGEs) in tissues increases in DM. AGEs can modify the functional properties of intracellular proteins such as antioxidant enzymes, induce crosslinking of collagen leading to increased stiffness of the blood vessels, and interact with a receptor for AGEs (RAGE), which activate proinflammatory signaling pathways [6,7,8]. Therefore, AGEs are thought to play an important role in the pathogenesis of diabetic microangiopathy [9,10,11], particularly in the progression of DR [12,13]. However, the relevance between AGEs levels and DR stages and the clinical utility of skin autofluorescence (sAF) measurements are largely unknown. 

In this article, we investigated the potential roles of AGEs during progression of DR. Since non-invasively measured sAF can easily estimate the levels of AGE accumulation, we examined the demographic differences and AGE scores measured by sAF in each DR stages and evaluated the clinical relevance of AGEs in patients with DM, especially those with proliferative diabetic retinopathy (PDR). We provided a novel approach to identify patients at risk for DR progression.

## 2. Materials and Methods

### 2.1. Subjects

The current study adhered to the tenets of the Declaration of Helsinki, and was approved by the institutional review boards of Matsue Red Cross Hospital and the Seirei Hamamatsu General Hospital. This study was conducted at the Matsue Red Cross Hospital (No. 303, issued on 21 September 2016), Iinan Hospital (No. 309, issued on 18 November 2016), and Seirei Hamamatsu General Hospital (No. 2198, issued on 20 July 2016). All participants gave written informed consent for inclusion in the study. All patient information was anonymized, and their identifiers were removed prior to analysis. All data were gathered by a non-blinded physician. Subjects were recruited consecutively at the outpatient clinics of the three hospitals.

We included 394 eyes of 394 Japanese subjects (172 men, 222 women; mean age ± standard deviation (SD), 68.4 ± 13.7 years), i.e., subjects with DM (*n* = 229) and non-diabetic controls (*n* = 165). In the patients with DM, if both eyes were eligible for inclusion in the study, the eye with the worse DR stage was included; if both eyes had the same stage, the eye with the worse BCVA was included; if both eyes had the same BCVA, the right eye was included. The control subjects were 20 years and older, had no history and clinical signs of DM, did not use glaucoma medications, and the previous highest IOP obtained by any type of tonometer was 20 mmHg or lower; the eyes with better BCVA were included in the analysis. Eyes with retinal diseases other than DR and those with glaucoma other than NVG were excluded from this study.

The subjects were interviewed about a history of DM, systemic hypertension, insulin use, and current smoking habits. In patients with DM, the most recent blood hemoglobin A1c (HbA1c) levels were collected during the chart reviews. The participants underwent examinations including measurement of the best-corrected visual acuity (BCVA), intraocular pressure (IOP) by Goldmann applanation tonometry (AT 900, Haag-Streit AG, Koeniz, Swizerland), slit-lamp microscopy (RO 5000, Rodenstock, Munich, Germany), gonioscopy using a two-mirror gonioscopy lens (Magna View Gonio, Ocular instruments, Washinton, DC, USA), fundoscopy using a non-contact lens (Super Field, Volk Optical, Mentor, USA), and fundus photograph images (Nonmyd WX, Kowa Company, Aichi, Japan). The lens status (phakia/pseudophakia) observed during slit-lamp examination and the previous highest IOPs measured by Goldmann applanation tonometry were obtained from the medical records.

The patients with DM were divided into groups based on those with no DR (NDR, *n* = 101) and DR (*n* = 128). DR included simple DR (SDR, *n* = 36), pre-proliferative DR (PPDR, *n* = 25), and PDR (*n* = 67). PDR was further divided into those with (*n* = 22) and without (*n* = 45) neovascular glaucoma (NVG). The DR stages were diagnosed based on bilateral funduscopic findings using the Davis classification of DR, specifically, the presence of retinal dot hemorrhages and hard exudations are signs of SDR; that of soft exudations, venous beading, and intraretinal microvascular abnormalities are signs of PPDR; and that of retinal neovascularization and vitreous or preretinal hemorrhages are signs of PDR [14]. NVG was diagnosed based on elevated IOP above 21 mmHg measured by Goldmann applanation tonometry and neovascularization of the iris and of the angle detected by slit lamp and gonioscopic examinations.

### 2.2. Measurement of AGEs in the Fingertip Skin

To estimate the AGEs, the participants underwent measurements of the sAF levels using the AGEs Sensor (Air Water Biodesign Inc., Kobe, Japan). The sAF levels were measured using the middle finger of the non-dominant hand in which the least skin melanin is present [15]. During the measurement, the fingertip was mildly compressed at the distal portion of the distal interphalangeal joint, which is the suitable region to avoid the non-specific sAF [16]. The sAF values were obtained with the excitation and emission wavelengths of 365 nm and 440 nm, respectively, which were correlated positively with the level of the hyperglycemia-associated AGEs, Nδ-(5-hydro-5-methyl-4-imidazolone-2-yl)-ornithine [16]. As we previously reported, the accuracy and usability of the AGEs Sensor was shown in the clinical settings [17]. The validation check process was carried out before each measuring time using an equipped fluorophore plate in the AGEs Sensor. The measurements of sAF were carried out consequently three times in each patient at the first visit, and the mean values were applied to the analyses. Trained examiners performed all measurements. Since the AGEs Sensor cannot measure accurately whether the finger to be measured has dirt on the surface of the skin, an injury, or a large amount of oil such as hand cream, removal of any dirt was conducted by using alcohol cotton. The measured AGEs were expressed as the AGE scores in arbitrary units with an upper limit of 10.0 and a lower limit of 0.0, and 0.5. An arbitrary unit approximately corresponds to the average scores of healthy Japanese subjects aged 50 years according to a recent manufacturer survey. The AGEs Sensor displays the value when the coefficient of variation reaches less than 1%. Based on our pilot study, the coefficient of variation and intraclass correlation coefficient (Cronbach’s α) of three repeated AGE measurements were 6.7 ± 7.3% and 0.938, respectively.

### 2.3. Statistical Analysis

To compare the two groups, the differences in the continuous data, i.e., age, BCVA, HbA1c, highest IOP, and AGE scores, were calculated using the unpaired Student *t*-test, and the differences in the categorical data, i.e., sex, lens status, presence of hypertension, insulin use, current smoking habit, and DR, were calculated using Fisher’s exact probability test. For comparisons among the three study groups, we performed one-way analysis of variance followed by post-hoc unpaired t-tests for continuous data and the G-test followed by the post-hoc Fisher’s exact probability test for categorical data. *P* values of 0.0167 and 0.0033 for the unpaired *t*-tests or Fisher’s exact probability test were considered significant levels at 5% and 1%, respectively, based on the Bonferroni correction. To determine the independent factors associated with the AGE score, we also performed multivariate logistic regression analyses with the quartiles of the AGE scores as the response variables and with the covariates of age, sex, lens status, BCVA, highest IOP, presence of hypertension, DR, and current smoking habit. All statistical analyses were calculated using the JMP Pro statistical software version 14.2 (SAS Institute, Inc., Cary, NC, USA). All reported *p* values are two-sided. The data are expressed as the means ± SD for continuous variables and as numbers and percentages for categorical variables. For the statistical analyses, the decimal BCVA was converted into the logarithm of the minimum angle of resolution (logMAR). Counting fingers, hand motions, light perception, and no light perception were regarded as decimal VAs of 0.0025, 0.002, 0.0016, and 0.0013, respectively [18].

## 3. Results

The demographic subject data, including age, sex, presence of hypertension, current smoking habit, insulin use, HbA1c, lens status, BCVA, highest IOP, and AGE scores, are shown in Table 1. All parameters differed significantly between the control and DM groups (*p* < 0.01), and all parameters except for hypertension were statistically significant among control, NDR, and DR groups (*p* < 0.05). Compared to the control group (0.52 ± 0.12), the AGE scores were significantly higher in the DM (0.59 ± 0.17, *p* < 0.0001), NDR (0.58 ± 0.16, *p* = 0.0012), and DR (0.60 ± 0.18, *p* < 0.0001) groups.

Table 2 shows the comparisons of groups stratified by quartiles of AGE scores. Significant differences were seen in the age, sex, DR, lens status, and highest IOP among the four quartiles (*p* < 0.05), and only the highest quartile (Q4) showed statistical significance compared with the Q1 and/or Q2 quartiles in post-hoc comparisons (*p* < 0.0083). The proportion of patients with PDR was significantly higher in the Q4 group than other quartiles (*p* < 0.0001).

The results of the comparison by multivariate logistic regression analysis of the Q1 and the Q2-Q4 groups are shown in Table 3. Multivariate analysis, which included age, sex, lens status, BCVA, highest IOP, presence of hypertension, DR, and current smoking habit, indicated that female gender (/men, OR = 0.38; *p* = 0.0059) and presence of DR = 0.0009, PDR (/control, OR = 6.73), non-PDR (/control, OR = 3.09) and PDR (/non-PDR, OR = 2.18) were independent variables significantly associated with the AGE score when compared to Q4 to Q1 as references. The results suggested that men and the presence of DM and DR were associated with the AGE score with the highest quartile. We also evaluated the association between AGEs scores and DR stages in male and female patients separately, which demonstrated statistically significant relevance between AGEs scores and DR grades only in female patients (Supplementary Table S1).

Since the DR was associated with a higher AGE level, we further compared the AGE level between subjects with PDR and other DR subjects (Table 4). The comparisons of demographic data according to DR grades were shown in Supplementary Table S2. Compared to the DM without the PDR group, the PDR group was younger (*p* = 0.0009) and more pseudophakic (*p* < 0.0001) and had worse BCVA (*p* < 0.0001) and higher IOP (*p* < 0.0001). Compared to the non-PDR group (0.56 ± 0.15), the AGE scores were significantly higher in the PDR group (0.64 ± 0.02, *p* = 0.0015); the difference was significant in PDR without NVG (0.64 ± 0.19, *p* = 0.0059) but was borderline in the PDR with NVG (0.64 ± 0.23, *p* = 0.0426).

Finally, we assessed the risk factors for PDR using multivariate models (Table 5). The results showed that, age (/year, OR = 0.92, *p* < 0.0001), female gender (/male gender, OR = 0.35, *p* = 0.0130), phakia (/pseudophakia, OR = 0.08, *p* < 0.0001), BCVA (/logMAR, OR = 23.67, *p* < 0.0001), highest IOP (/mmHg, OR = 1.08, *p* = 0.0197), and AGE scores (/A.U., OR = 27.85, *p* = 0.0100) were associated significantly with PDR, although hypertension and current smoking were not. The results suggested that younger age, male gender, pseudophakia, worse VA, higher IOP, and higher AGEs scores were the risk factors for PDR.

## 4. Discussion

This study was designed to investigate the role of AGEs in patients with DR. Overall, the results suggested two important clinical findings. First, AGE scores were higher in patients with DM, and independently associated with progression of DR. Second, the highest quartile of the AGE scores had a higher proportion of PDR than the other quartiles.

The current results showed that AGEs were an independent factor for development of DR. In fact, the AGE scores were significantly higher in patients with DR and those with DM compared to the control group, and the higher AGE score was correlated with the progression of DR. In addition, AGEs were an independent risk factor for PDR; multivariate analyses were performed to adjust for background characteristics. Previous reports have shown that sAF was well correlated with tissue accumulation of AGEs, past glycemic control in patients with DM [13], and the severity of DR [19]. The results of our study were consistent with those of previous studies, and the evidence we report suggested that AGEs would be a key exacerbating factor for the progression of DR.

One possible explanation for this is that AGEs play an important role in the oxidative stress-induced apoptosis of the retinal capillary pericytes [20]. Several studies have elucidated that AGEs can induce intrinsic signaling pathways mediated mainly through a receptor for AGEs (RAGE) expressed on the membrane of pericytes, leading to apoptosis of pericytes [21,22,23]. Since pericytic function is the main regulator of the basement membrane at the blood retinal barrier [24], selective pericyte loss, induced by AGEs-RAGE signaling pathways, leads to disruption of the blood retinal barrier and the following development of DR [25]. Therefore, it is biologically plausible that AGEs contribute to the progression of DR.

The second clinical suggestion we provide here is that the highest quartile of the AGE scores was associated with a higher proportion of DR, especially PDR. Previous reports have shown a clear correlation between the prevalence of DR and serum hydroimidazolone levels, which is one of the most abundant AGEs in vivo, and the highest levels of hydroimidazolone in patients with PDR [26]. This evidence is particularly consistent with the current results. It is important that only the highest quartile group had a significantly higher proportion of PDR. This observation suggested that the level of AGEs might not be correlated linearly with the severity of the DR stages; AGEs might have been physiologically significant in the development of PDR when the levels of AGEs become markedly elevated.

A possible mechanism to explain this finding is that AGEs can promote intraocular vascular proliferation and inflammatory response, mainly through production of vascular endothelial growth factor (VEGF) and proinflammatory cytokines such as interleukins and tumor necrosis factors [27,28,29,30]. Previous reports have shown that AGEs stimulate release of VEGF by pericytes and Müller cells [28,31], which is conventionally recognized as a key physiologic mechanism of ischemia-induced retinal neovascularization [32]. In addition, a number of studies have revealed that inflammation is a critical contributor for the development of PDR [33], and AGEs can initiate inflammatory process via RAGE-mediated NF-κB signaling pathways [34]. These evidences strongly support our hypothesis that accumulation of AGEs to markedly high levels (i.e., the top 25 percentile) would be deeply involved in the development of PDR.

It is important to emphasize that our research showed no statistically significant difference of AGEs score between PDR and NVG groups. The pathogenesis of NVG is a neovascularization associated with retinal ischemia and ocular inflammatory response, however the majority of cases are related to excess VEGF production following retinal ischemia [35]. Therefore, we hypothesized that ischemia-induced VEGF production would be a much stronger aggravating factor for the development of NVG than the inflammatory response via RAGE signaling pathways. In fact, a previous study reported that the VEGF concentration in the vitreous fluid from patients with active PDR was significantly higher than in patients without PDR [36]. Nevertheless, we did not evaluate the associations between VEGF concentration and AGEs levels, and this hypothesis needs further investigation.

It is also interesting to note that younger age, male gender, pseudophakia, worse VA, and higher IOP were associated significantly with PDR in this study. This finding appears to be partially consistent with previously reported results, which indicated the independent risk factors for PDR [37,38]. Interestingly, our research revealed that younger age and male gender were associated with higher AGEs scores. The difference of dietary AGEs might explain this observation [39]. In addition, we also showed that pseudophakia was a significant risk factor for PDR [40,41]; a possible hypothesis for this finding is that AGEs might have an important role in the pathogenesis of cataracts. A previous report suggested that this hypothesis showed that the serum AGEs in diabetic patients with cataracts were significantly higher than those in non-diabetic controls without cataracts [42]. The current study reported the baseline characteristics of patients with PDR, which could be informative to identify individuals at risk of the development of PDR.

The current study had some limitations that might affect the generalizability of our findings. The complete demographic data were unavailable, which creates a potential selection bias. We did not directly measure serum AGEs concentration, however the fluorescence intensity measured by the AGEs Sensor was well correlated to the serum AGEs concentrations, and the increase of fluorescence intensity was associated with the presence of diabetic microvascular complications [16]. In addition, the measurement of AGEs via sAF was affected by skin pigmentation, which leads to lower measurement accuracy [15]. Nevertheless, the fingertip skin-based measurement with the AGEs Sensor used in this study might compensate for this shortage [17]. Therefore, the non-invasive measurement of skin AGEs accumulation by the AGEs Sensor was thought to be a reasonable method for the current study. Despite these limitations, our study has many strengths, including the large sample size of individuals, non-invasive objective measurement of AGEs, and comprehensive assessments of patients’ clinical characteristics.

## 5. Conclusions

The current results suggested that AGE scores were higher in patients with DM and independently associated with the progression of DR. In addition, the highest quartile of AGE scores had a higher proportion of patients with DR, in particular PDR. This study highlights the clinical use of the AGE score as a non-invasive and reliable marker of patients at risk of sight-threatening DR. The current findings warrant further research to identify the optimal cut-off value of the AGE score for differentiating patients with a high risk of developing severe DR.

## Figures and Tables

**Table 1:** Demographic subject data.

Parameters	Control	DM	*p*-Value ^a^	□	NDR	DR	*p*-Value ^b^
N	165	229			101	128	
Age (years)							
Mean ± SD	70.9 ± 14.1	66.6 ± 13.0	0.0017 **		68.0 ± 14.6	65.5 ± 11.6	0.0028 **
range	23–95	16–95			16–95	32–92	
				*p*-value, vs. Control ^c^	0.0845	0.0007 ^##^	
				*p*-value, vs. NDR ^c^	−	0.1638	
Sex							
Men, n (%)	47 (28.5)	125 (54.6)	<0.0001 **		49 (48.5)	76 (59.4)	<0.0001 **
Women, n (%)	118 (71.5)	104 (45.4)			52 (51.5)	52 (40.6)	
				*p*-value, vs. Control ^c^	0.0057 ^##^	<0.0001 ^##^	
				*p*-value, vs. NDR ^c^	−	0.1102	
Hypertension							
No, n (%)	94 (57.0)	105 (45.9)	0.0323 *		49 (48.5)	56 (43.8)	0.0723
Yes, n (%)	71 (43.0)	124 (54.1)			52 (51.5)	72 (56.2)	
HbA1c (%)							
Mean ± SD	−	7.8 ± 0.1	−		8.1 ± 2.7	7.6 ± 1.8	-
range	−	4.4–17.3			4.4–17.3	5.6–16.0	
Insulin usage							
No, n (%)	−	144 (63.7)	−		71 (72.4)	73 (57.0)	-
Yes, n (%)	−	82 (36.3)			27 (27.6)	55 (43.0)	
Current smoking habit							
No, n (%)	153 (92.7)	189 (82.9)	0.0039 **		82 (81.2)	107 (84.2)	0.0132 *
Yes, n (%)	12 (7.3)	39 (17.1)			19 (18.8)	20 (15.8)	
				*p*-value, vs. Control ^c^	0.0057 ^##^	0.0242 ^#^	
				*p*-value, vs. NDR ^c^	-	0.5971	
Lens status							
Phakic, n (%)	132 (80.0)	143 (62.5)	0.0002 **		79 (78.2)	64 (50.0)	<0.0001 **
Pseudophakic, n (%)	33 (20.0)	87 (37.5)			22 (21.8)	64 (50.0)	
				*p*-value, vs. Control^c^	0.7564	<0.0001 ^##^	
				*p*-value, vs. NDR^c^	−	<0.0001 ^##^	
BCVA (LogMAR)							
Mean ± SD	0.12 ± 0.22	0.34 ± 0.65	<0.0001 **		0.10 ± 0.27	0.53 ± 0.78	<0.0001 **
range	−0.08–1.40	−0.08–2.89			−0.08–1.30	−0.08–2.89	
				*p*-value, vs. Control ^c^	0.7673	<0.0001 ^##^	
				*p*-value, vs. NDR ^c^	−	<0.0001 ^##^	
Highest IOP (mmHg)							
Mean ± SD	14.3 ± 2.8	19.2 ± 9.5	<0.0001 **		16.5 ± 4.3	21.4 ± 11.7	<0.0001 **
range	6.9–20.0	8.0–80.0			9.7–35.0	8.0–80.0	
				*p*-value, vs. Control ^c^	0.0173 ^#^	<0.0001 ^##^	
				*p*-value, vs. NDR ^c^	−	<0.0001 ^##^	
AGEs score (A.U.)							
Mean ± SD	0.52 ± 0.12	0.59 ± 0.17	<0.0001 **		0.58 ± 0.16	0.60 ± 0.18	<0.0001 **
range	0.26–1.21	0.25–1.55			0.25–1.48	0.26–1.55	
				*p*-value, vs. Control ^c^	0.0012 ^##^	<0.0001 ^##^	
				*p*-value, vs. NDR ^c^	−	0.3349	

^a^ Comparison between control and DM groups by using unpaired Student *t*-test for continuous data and by using Fisher’s exact probability test for categorical data. * and ** correspond to the significance levels at 5% (*p* < 0.05) and 1% (*p* < 0.01), respectively. ^b^ Comparison among control, NDR, and DR groups by using one-way ANOVA for continuous data and by using G-test for categorical data. ^c^ Comparison between either pair of control, NDR, or DR groups by using post-hoc unpaired Student *t*-test for continuous data and by using Fisher’s exact probability test for categorical data. # and ## correspond to the significance levels at 5% (*p* < 0.0167) and 1% (*p* < 0.0033), respectively, by Bonferroni correction for multiple comparisons. One aphakic case is included in pseudophakic cases. N, number of participants; SD, standard deviation; DM, diabetes mellitus; NDR, no diabetic retinopathy; DR, diabetic retinopathy; Glycated Hemoglobin A1c; BCVA, best-corrected visual acuity; IOP, intraocular pressure; AGEs, advanced glycation end products; A.U., arbitrary unit.

**Table 2:** Demographic subject data stratified by quartiles of AGE scores.

Parameters	Q1	Q2	Q3	Q4	*p*-Value ^a^
Range	Low–≤0.467	>0.467–≤0.539	>0.539–≤0.635	>0.635–High	
N					
Age (years)					
Mean ± SD	71.4 ± 12.3	68.1 ± 12.9	68.5 ± 13.8	65.5 ± 15.0	0.0259 *
Range	16–95	23–92	23–95	28–94	
*p*-value, vs. Q1 ^b^	−	0.0903	0.1271	0.0024 ^#^	
*p*-value, vs. Q2 ^b^	−	−	0.8571	0.1851	
*p*-value, vs. Q3 ^b^	−	−	−	0.1296	
Sex					
Men, n (%)	34 (33.7)	40 (41.4)	41 (41.4)	57 (58.2)	0.0011 **
Women, n (%)	67 (66.3)	56 (58.3)	58 (58.6)	41 (41.8)	
*p*-value, vs. Q1 ^b^	−	0.3030	0.3069	0.0006 ^##^	
*p*-value, vs. Q2 ^b^	−	−	1.0000	0.0310	
*p*-value, vs. Q3 ^b^	−	−	−	0.0227	
Hypertension					
No, n (%)	52 (51.5)	55 (57.3)	50 (50.5)	42 (42.9)	0.1647
Yes, n (%)	49 (48.5)	41 (42.7)	49 (49.5)	56 (57.1)	
Diabetic retinopathy					
Control, n (%)	55 (54.5)	45 (46.9)	47 (47.5)	18 (18.4)	<0.0001 **
NDR, n (%)	24 (23.8)	23 (24.0)	24 (24.2)	30 (30.6)	
SDR, n (%)	7 (6.9)	7 (7.3)	11 (11.1)	11 (11.2)	
PPDR, n (%)	7 (6.9)	9 (9.4)	3 (3.0)	6 (6.1)	
PDR, n (%)	8 (7.9)	12 (12.5)	14 (14.1)	33 (33.7)	
HbA1c (%)					
Mean ± SD	7.5 ± 1.2	8.0 ± 2.2	7.8 ± 2.3	7.9 ± 2.3	0.6009
Range	5.6–10.8	5.6–14.6	5.8–17.3	4.4–16	
Insulin use					
No, n (%)	30 (66.7)	38 (74.5)	33 (66.0)	43 (53.8)	0.0530
Yes, n (%)	15 (33.3)	13 (25.5)	17 (34.0)	37 (46.3)	
Current smoking habit					
No, n (%)	86 (85.1)	82 (86.3)	90 (90.9)	84 (85.7)	0.6899
Yes, n (%)	15 (14.9)	13 (13.7)	9 (9.1)	14 (14.3)	
Lens status					
Phakic, n (%)	76 (75.3)	70 (72.9)	72 (72.7)	58 (59.2)	0.0213 *
Pseudophakic, n (%)	25 (24.8)	26 (27.1)	27 (27.3)	40 (40.9)	
*p*-value, vs. Q1 ^b^	−	0.7466	0.7480	0.0230	
*p*-value, vs. Q2 ^b^	−	−	1.0000	0.0496	
*p*-value, vs. Q3 ^b^	−	−	−	0.0513	
BCVA (logMAR)					
Mean ± SD	0.19 ± 0.39	0.23 ± 0.47	0.23 ± 0.54	0.33 ± 0.66	0.3040
Range	−0.08–2.89	−0.08–2.89	−0.08–2.89	−0.08–2.89	
Highest IOP (mmHg)					
Mean ± SD	15.4 ± 5.1	16.0 ± 6.0	17.8 ± 8.3	19.6–0.78	0.0006 **
Range	6.9–44	10–63	8–68	9–80	
*p*-value, vs. Q1 ^b^	−	0.5748	0.0286	0.0001 ^##^	
*p*-value, vs. Q2 ^b^	−	−	0.1082	0.0013 ^##^	
*p*-value, vs. Q3 ^b^	−	−	−	0.1003	

^a^ Comparison of characteristics stratified by the quartiles of the AGE scores using one-way analysis of variance for continuous data and using the exact Cochran-Armitage trend test or G-test for categorical data. * and ** correspond to the significance levels at 5% (*p* < 0.05) and 1% (*p* < 0.01), respectively. ^b^ Comparison between either pair of Q1-4 groups using the post-hoc unpaired Student *t*-test for continuous data and using Fisher’s exact probability test for categorical data. # and ## correspond to the significance levels at 5% (*p* < 0.0083) and 1% (*p* < 0.0016), respectively, using the Bonferroni correction for multiple comparisons. One aphakic case is included among the pseudophakic cases. Q, quartile; N, number of participants; SD, standard deviation; NDR, no diabetic retinopathy; SDR, simple diabetic retinopathy; PPDR, pre-proliferative diabetic retinopathy; PDR, proliferative diabetic retinopathy; HbA1c, glycated hemoglobin A1c; BCVA, best-corrected visual acuity; IOP, intraocular pressure; logMAR, logarithm of the minimum angle of resolution.

**Table 3:** Multivariate logistic regression analysis for comparisons among quartile AGEs groups.

Parameters	Q1	Q2	Q3	Q4
Entire model				
*p*-value	—	0.0001 **	0.0005 **	<0.0001 **
Age (/year)				
*p*-value	—	0.0981	0.1670	0.0540
OR (95% CI)	1	0.98 (0.95–1.00)	0.98 (0.96–1.01)	1.03 (0.99–1.05)
Women (/men)				
*p*-value	—	0.2552	0.1668	0.0059 **
OR (95% CI) women/men	1	0.68 (0.35–1.32)	0.62 (0.33–1.21)	0.38 (0.20–0.76) **
Hypertension, yes (/no)				
*p*-value	—	0.6611	0.5677	0.1388
OR (95% CI)	1	0.87 (0.48–1.59)	1.19 (0.64–2.24)	1.64 (0.85–3.18)
DR, yes (/no)				
*p*-value		0.9521	0.9547	0.0009 **
OR (95% CI) non-PDR/control		1.11 (0.58–2.11)	0.95 (0.45–2.00)	3.09 (1.48–6.44) **
OR (95% CI) PDR/control		1.08 (0.31–3.72)	1.10 (0.31–3.83)	6.73 (1.96–23.16) **
OR (95% CI) PDR/non-PDR		0.98 (0.30–3.16)	1.17 (0.35–3.92)	2.18 (0.68–6.96)
Current smoking habit, yes(/no)				
*p*-value	—	0.3262	0.0198 *	0.1309
OR (95% CI)	1	0.64 (0.26–1.58)	0.29 (0.10–0.82) *	0.48 (0.18–1.25)
Phakic (/pseudophakic)				
*p* value	—	0.4358	0.5659	0.3425
OR (95% CI)	1	0.75 (0.36–1.56)	1.26 (0.57–2.77)	0.67 (0.29–1.53)
BCVA (/logMAR)				
*p*-value	—	0.5757	0.4068	0.7487
OR (95% CI)	1	1.24 (0.56–2.85)	0.70 (0.31–1.62)	0.67 (0.29–1.53)
Highest IOP (/mmHg)				
*p*-value	—	0.9206	0.0097 **	0.6988
OR (95% CI)	1	1.00 (0.93–1.06)	1.09 (1.01–1.16) **	0.99 (0.93–1.05)

Data analysis was performed using multivariate logistic regression analysis to compare the Q1 and the Q2-4 groups. Independent variables considered for the model are those thought to be possibly biologically related to the AGE score. DR grades are classified into three groups including PDR, non-PDR (DM without PDR), and control. *P* values were calculated using the likelihood ratio test. * and ** correspond to the significance levels at 5% (*p* < 0.05) and 1% (*p* < 0.01), respectively. To calculate the odds ratios, the Q1 group is the reference. Q, quartile; OR, odds ratio; CI, confidence interval; y, yes; n, no; DR, diabetic retinopathy; PDR, no diabetic retinopathy; BCVA, best-corrected visual acuity; IOP, intraocular pressure; OR, odds ratio.

**Table 4:** Demographic data of DR subjects stratified by presence/absence of PDR.

Parameters	Non-PDR	PDR	*p*-Value ^a^	□	PDR, non-NVG	PDR, NVG	*p*-Value ^b^
N	162	67			45	22	
Age (years)							
Mean ± SD	68.4 ± 13.2	62.2 ± 11.6	0.0009 **		62.0 ± 11.8	62.5 ± 11.5	0.0039 **
Range	16–95	32–83			32–82	38–83	
				*p*-value, v.s. no PDR^c^	0.0033 ^#^	0.0411	
				*p*-value, v.s. PDR^c^	—	0.8967	
Sex							
Men, n (%)	84 (51.9)	41 (61.2)	0.2433		29 (64.4)	12 (54.6)	0.3242
Women, n (%)	78 (48.1)	26 (38.8)			16 (35.6)	10 (45.5)	
Hypertension							
No, n (%)	76 (46.9)	29 (43.3)	0.6633		21 (46.7)	8 (36.4)	0.6430
Yes, n (%)	86 (53.1)	38 (56.7)			24 (53.3)	14 (63.6)	
HbA1c (%)							
Mean ± SD	8.0 ± 2.2	7.4 ± 1.8	0.0313 *		7.6 ± 0.3	7.2 ± 1.5	0.1369
range	4.4–17.3	5.6–16			5.9–16.0	5.6–11.6	
Insulin usage							
No, n (%)	107 (67.3)	37 (55.2)	0.0966		28 (62.2)	9 (40.9)	0.0530
Yes, n (%)	52 (32.7)	30 (44.8)			17 (37.8)	13 (50.1)	
Current smoking habit							
No, n (%)	132 (82.0)	57 (85.1)	0.7002		39 (86.7)	18 (81.8)	0.7547
Yes, n (%)	29 (18.0)	10 (16.7)			6 (13.3)	4 (18.2)	
Lens status							
Phakic, n (%)	121 (74.7)	23 (34.3)	<0.0001 **		19 (42.2)	4 (18.2)	<0.0001 **
Pseudophakic, n (%)	41 (25.3)	44 (65.7)			26 (57.8)	18 (81.8)	
				*p*-value, v.s. no PDR ^c^	0.0001 ^##^	<0.0001 ^##^	
				*p*-value, v.s. PDR ^c^	−	0.0606	
BCVA (LogMAR)							
Mean ± SD	0.14 ± 28	0.83 ± 0.95	<0.0001 **		0.74 ± 0.86	1.01 ± 1.11	<0.0001 **
range	−0.07–2.88	−0.08–2.89			−0.08–2.89	−0.08–2.89	
				*p*-value, v.s. no PDR ^c^	0.0002 ^##^	<0.0001 ^##^	
				*p*-value, v.s. PDR ^c^	−	0.1614	
Highest IOP (mmHg)							
Mean ± SD	16.6 ± 4.1	25.7 ± 1.3	<0.0001 **		19.9 ± 8.6	37.5 ± 17.3	<0.0001 **
range	9–35	8–80			8–51	17–80	
				*p*-value, v.s. no PDR ^c^	<0.0001 ^##^	<0.0001 ^##^	
				*p*-value, v.s. PDR ^c^	−	0.0748	
AGEs score (A.U.)							
Mean ± SD	0.56 ± 0.15	0.64 ± 0.02	0.0015 *		0.64 ± 0.19	0.64 ± 0.23	0.0064 **
range	0.25–1.48	0.39–1.55			0.40–1.53	0.39–1.55	
				*p*-value, v.s. no PDR ^c^	0.0059 ^#^	0.0426	
				*p*-value, v.s. PDR ^c^	−	0.9852	

^a^ Comparison between non-PDR (DM without PDR) and PDR groups by using unpaired Student *t*-test for continuous data and by using Fisher’s exact probability test for categorical data. The * and ** correspond to the significance levels at 5% (*p* < 0.05) and 1% (*p* < 0.01), respectively. ^b^ Comparison among non-PDR, PDR without NVG, and PDR with NVG groups by using one-way ANOVA for continuous data and by using G-test for categorical data. ^c^ Comparison between either pairs of non-PDR, PDR without NVG, or PDR with NVG groups by using post-hoc unpaired Student *t*-test for continuous data and by using Fisher’s exact probability test for categorical data. The # and ## correspond to the significance levels at 5% (*p* < 0.0167) and 1% (*p* < 0.0033), respectively, by Bonferroni correction for multiple comparisons. One aphakic case is included in pseudophakic cases. N, number of participants; SD, standard deviation; SDR, simple diabetic retinopathy; PPDR, pre-proliferative diabetic retinopathy; PDR, proliferative diabetic retinopathy; NVG, neovascular glaucoma; HbA1c, Glycated Hemoglobin A1c; BCVA, best-corrected visual acuity; IOP, intraocular pressure; AGEs, advanced glycation end products; A.U., arbitrary unit.

**Table 5:** Multivariate logistic regression analysis for risk factors of PDR.

Parameters	OR	95% CI	*p*-Value ^a^
Entire model	—	—	<0.0001 **
Age (/years)	0.92 **	0.89–0.95	<0.0001 **
Women(/men)	0.35 *	0.15–0.81	0.0130 *
Hypertension, yes (/no)	1.20	0.54–2.66	0.6629
Current smoking habit, yes (/no)	0.48	0.13–1.79	0.2613
Phakic (/pseudophakic)	0.08 **	0.03–0.22	<0.0001 **
BCVA (/logMAR)	23.67 **	7.40–75.75	<0.0001 **
Highest IOP (/mmHg)	1.08 *	1.01–1.16	0.0197 *
AGE score (/A.U.)	27.85 *	2.69–288.30	0.0100 *

Multivariate logistic regression analysis was performed with the following factors: age, sex, hypertension, current smoking habit, lens status, VA, highest IOP, and AGE score. ^a^ *p* values were calculated using the likelihood ratio test. * and ** correspond to the significance levels at 5% (*p* < 0.05) and 1% (*p* < 0.01), respectively. OR, odds ratio; CI, confidence interval; y, yes; n, no; BCVA, best-corrected visual acuity; IOP, intraocular pressure; AGEs, advanced glycation end products; A.U., arbitrary units; logMAR, logarithm of the minimum angle of = resolution; BCVA, best-corrected visual acuity.

## Supplementary Materials

The following are available online at https://www.mdpi.com/2076-3921/9/11/1100/s1. Supplementary Table S1. Association between AGEs score and diabetic retinopathy grades by gender. Supplementary Table S2. Demographic subject data according to diabetic retinopathy grades.
