# Supplementary material for: Evaluation of Relevance between Advanced Glycation End Products and Diabetic Retinopathy Stages Using Skin Autofluorescence
Source: Antioxidants (Basel). 2020 Nov 9;9(11):1100. doi: 10.3390/antiox9111100 (PMC7695256; doi:10.3390/antiox9111100)
Supplement: Supplementary file 1 [file antioxidants-09-01100-s001.pdf]

Supplementary Table 1. Association between AGEs score and diabetic retinopathy grades by gender

	Q1	Q2	Q3	Q4	p-value[a]
range	Low - ≤ 0.467	> 0.467 - ≤ 0.539	> 0.539 - ≤ 0.635	> 0.635 - High	
Men, n (%)	34 (19.8)	40 (23.3)	41 (23.8)	57 (33.1)	
Diabetic retinopathy					0.1866
Control, n (%)	13 (38.2)	12 (30.0)	13 (31.7)	9 (19.2)	
NDR, n (%)	10 (29.4)	9 (22.5)	10 (24.4)	20 (40.8)	
SDR, n (%)	4 (11.8)	4 (10.0)	7 (17.7)	7 (31.8)	
PPDR, n (%)	3 (8.8)	6 (15.0)	2 (4.88)	2 (15.4)	
PDR, n (%)	4 (9.8)	9 (22.5)	9 (22.0)	19 (46.3)	

	Q1	Q2	Q3	Q4	p-value[a]
range	Low - ≤ 0.467	> 0.467 - ≤ 0.539	> 0.539 - ≤ 0.635	> 0.635 - High	
Women, N	67 (30.2)	56 (25.2)	58 (26.1)	41 (18.5)	
Diabetic retinopathy					0.0004**
Control, n (%)	42 (62.7)	33 (58.9)	34 (58.6)	9 (22.0)	
NDR, n (%)	14 (20.9)	14 (25.0)	14 (24.1)	10 (24.4)	
SDR, n (%)	3 (4.5)	3 (5.4)	4 (6.9)	4 (9.8)	
PPDR, n (%)	4 (6.0)	3 (5.4)	1 (1.7)	4 (9.8)	
PDR, n (%)	4 (6.0)	3 (5.4)	5 (8.6)	14 (53.9)	

[a]Comparison of characteristic stratified by quartiles of AGEs score by using G-test for categorical data. The * and ** correspond to the signifcance levels at 5% (P < 0.05) and 1% (P < 0.01), respectively. Q, quartile; N, number of participants; NDR, no diabetic retinopathy; SDR, simple diabetic retinopathy; PPDR, pre-proliferative diabetic retinopathy; PDR, proliferative diabetic retinopathy.

Supplementary Table 2. Demographic subject data according to diabetic retinopathy grades

	CT	NDR	SDR	PPDR	PDR	p value[a]
N	165	101	36	25	67	
Age (years)						
Mean ± SD	70.9 ± 14.1	68.0 ± 14.6	69.2 ± 9.9	69.1 ± 11.7	62.2 ± 11.6	0.0004**
range	23-95	16-95	40-83	42-92	32-83	
p-value, v.s. CT[b]	–	0.0819	0.4719	0.5177	<0.0001##	
p-value, v.s. NDR[b]	–	–	0.6508	0.7156	0.0060	
p-value, v.s. SDR[b]	–	–	–	0.9802	0.0117	
p-value, v.s. PPDR[b]	–	–	–	–	0.0280	
Sex						
Men, n (%)	47 (28.5)	49 (48.5)	22 (61.1)	13 (52.0)	41 (61.2)	<0.0001**
Women, n (%)	118 (71.5)	52 (51.5)	14 (38.9)	12 (48.0)	26 (38.8)	
p-value, v.s. CT[b]	–	0.0015#	0.0004	0.0355	<0.0001##	
p-value, v.s. NDR[b]	–	–	0.2446	0.8251	0.1167	
p-value, v.s. SDR[b]	–	–	–	0.6001	1.000	
p-value, v.s. PPDR[b]	–	–	–	–	0.4799	
Hypertension						
No, n (%)	94 (57.0)	49 (48.5)	17 (47.2)	10 (40.0)	29(43.3)	0.0324*
Yes, n (%)	71 (43.0)	52 (51.5)	19 (52.8)	15 (60.0)	38 (56.7)	
p-value, v.s. CT[b]	–	0.2056	0.3555	0.1334	0.0612	
p-value, v.s. NDR[b]	–	–	1.000	0.5065	0.5309	
p-value, v.s. SDR[b]	–	–	–	0.6103	0.8356	
p-value, v.s. PPDR[b]	–	–	–	–	0.8167	
HbA1c (%)						
Mean ± SD	—	8.1 ± 2.7	8.0 ± 1.8	7.6 ± 1.7	7.4 ± 1.8	0.2025
range	—	4.4-17.3	5.8-12.6	5.7-12.2	5.6-16.0	
Insulin usage						
Yes, n (%)	—	27 (27.6)	16 (44.4)	9 (36.0)	30 (44.8)	0.0363*
No, n (%)	—	71 (72.4)	20 (55.6)	16 (64.0)	37 (55.2)	
p-value, v.s. CT[b]	–	–	–	–	–	
p-value, v.s. NDR[b]	–	–	0.0938	0.4626	0.0300	
p-value, v.s. SDR[b]	–	–	–	0.6007	1.000	
p-value, v.s. PPDR[b]	–	–	–	–	0.4864	
Current smoking habit						
Yes, n (%)	12 (7.3)	19 (18.8)	7 (20.0)	3 (12.0)	10 (14.9)	0.1297
No, n (%)	153 (92.7)	82 (81.2)	28 (80.0)	22 (88.0)	57 (85.1)	
Phakia						
Phakic, n (%)	132 (80.0)	79 (78.2)	27 (75.0)	15 (60.0)	22 (32.8)	<0.0001**
Pseudophakic, n (%)	33 (20.0)	22 (21.8)	9 (25.0)	10 (40.0)	45 (67.2)	
p-value, v.s. CT[b]	–	0.7564	0.5025	0.0383	<0.0001##	
p-value, v.s. NDR[b]	–	–	0.8168	0.0745	<0.0001##	
p-value, v.s. SDR[b]	–	–	–	0.2659	<0.0001##	
p-value, v.s. PPDR[b]	–	–	–	–	0.0333	
VA (LogMAR)						
Mean ± SD	0.12 ± 0.22	0.10 ± 0.27	0.13 ± 0.25	0.30 ± 0.31	0.83 ± 0.95	<0.0001**
range	-0.08 - 1.40	-0.08 - 1.30	-0.08 - 1.10	-0.08 - 1.00	-0.08 - 2.88	
p-value, v.s. CT[b]	–	0.7499	0.8900	0.0644	<0.0001##	
p-value, v.s. NDR[b]	–	–	0.7350	0.0505	<0.0001##	
p-value, v.s. SDR[b]	–	–	–	0.1532	<0.0001##	
p-value, v.s. PPDR[b]	–	–	–	–	<0.0001##	
Highest IOP (mmHg)						
Mean ± SD	14.3 ± 2.8	16.5 ± 4.3	17.0 ± 4.2	16.1 ± 3.0	25.7 ± 14.6	<0.0001**
range	6.9 - 20.0	9.7 - 35.0	9.0 - 27.0	10.7 - 22.0	8.0 - 80.0	
p-value, v.s. CT[b]	–	0.0112	0.0341	0.2192	<0.0001##	
p-value, v.s. NDR[b]	–	–	0.7220	0.7953	<0.0001##	
p-value, v.s. SDR[b]	–	–	–	0.6257	<0.0001##	
p-value, v.s. PPDR[b]	–	–	–	–	<0.0001##	
AGEs score (A.U.)						
Mean ± SD	0.52 ± 0.12	0.58 ± 0.16	0.56 ± 0.13	0.52 ± 0.14	0.64 ± 0.20	<0.0001**
range	0.26 - 1.21	0.25 - 1.48	0.30 - 0.81	0.26 - 0.86	0.39 - 1.55	
p-value, v.s. CT[b]	–	0.0010#	0.0850	0.8137	<0.0001##	
p-value, v.s. NDR[b]	–	–	0.6074	0.1014	0.0055	
p-value, v.s. SDR[b]	–	–	–	0.3057	0.0094	
p-value, v.s. PPDR[b]	–	–	–	–	0.0006#	

[a]Comparison among CT, SDR, DMR, PPDR, and PDR groups by using one-way ANOVA for continuous data and by exact Cochran-Armitage trend test for categorical data. The * and ** correspond to the signifcance levels at 5% (P < 0.05) and 1% (P < 0.01), respectively. [b]Comparison between either pairs of CT, NDR, DMR, PPDR, or PDR groups by using post-hoc unpaired Student t-test for continuous data and by using Fisher's exact probability test for categorical data. The # and ## correspond to the signifcance levels at 5% (P < 0.005) and 1% (P < 0.001), respectively, by Bonferroni correction for multiple comparisons, and only those p values that reached statistical significance are indicated. One aphakic case was included in pseudophakic cases. N, number of participants; SD, standard deviation; CT, control; NDR, no diabetic retinopathy; SDR, simple diabetic retinopathy; PPDR, pre-proliferative diabetic retinopathy; PDR, proliferative diabetic retinopathy; HbA1c, Glycated Hemoglobin A1c; VA, visual acuity; IOP, intraocular pressure.
